# Development and validation of natural language processing algorithms in the national ENACT network

**DOI:** 10.1017/cts.2025.10116

**Published:** 2025-08-22

**Authors:** Yanshan Wang, Jordan Hilsman, Chenyu Li, Michele Morris, Paul M. Heider, Sunyang Fu, Min Ji Kwak, Andrew Wen, Joseph R. Applegate, Liwei Wang, Elmer Bernstam, Hongfang Liu, Jack Chang, Daniel R. Harris, Alexandria Corbeau, Darren Henderson, John Osborne, Richard E. Kennedy, Nelly-Estefanie Garduno-Rapp, Justin F. Rousseau, Chao Yan, You Chen, Mayur B. Patel, Tyler J. Murphy, Bradley A. Malin, Chan Mi Park, Jungwei W. Fan, Sunghwan Sohn, Sandeep Pagali, Yifan Peng, Aman Pathak, Yonghui Wu, Zongqi Xia, Salvatore Loguercio, Steven E. Reis, Shyam Visweswaran

**Affiliations:** 1 Clinical and Translational Science Institute, University of Pittsburgh, Pittsburgh, PA, USA; 2 Department of Health Information Management, University of Pittsburgh, Pittsburgh, PA, USA; 3 Department of Biomedical Informatics, University of Pittsburgh, Pittsburgh, PA, USA; 4 Biomedical Informatics Center and Department of Public Health Sciences, Medical University of South Carolina, Charleston, SC, USA; 5 McWilliams School of Biomedical Informatics, University of Texas Health Science Center at Houston, Houston, TX, USA; 6 McGovern Medical School, University of Texas Health Science Center at Houston, Houston, TX, USA; 7 Division of General Internal Medicine, McGovern Medical School, University of Texas Health Science Center at Houston, Houston, TX, USA; 8 Clinical and Translational Science Institute, University of Rochester Medical Center, Rochester, NY, USA; 9 Institute for Biomedical Informatics, University of Kentucky, Lexington, KY, USA; 10 Department of Biomedical Informatics and Data Science, University of Alabama at Birmingham, Birmingham, AL, USA; 11 Division of Gerontology, Geriatrics, and Palliative Care, Department of Medicine, University of Alabama at Birmingham, Birmingham, AL, USA; 12 Clinical Informatics Center, University of Texas Southwestern Medical Center, Dallas, TX, USA; 13 Department of Neurology, University of Texas Southwestern Medical Center, Dallas, TX, USA; 14 Department of Biomedical Informatics, Vanderbilt University Medical Center, Nashville, TN, USA; 15 Department of Surgery, Vanderbilt University Medical Center, Nashville, TN, USA; 16 Department of Gerontology, Hebrew SeniorLife, Marcus Institute for Aging Research, Boston, MA, USA; 17 Department of Artificial Intelligence and Informatics, Mayo Clinic, Rochester, MN, USA; 18 Center for Clinical and Translational Science, Mayo Clinic, Rochester, MN, USA; 19 Department of Medicine, Mayo Clinic, Rochester, MN, USA; 20 Department of Population Health Sciences, Weill Cornell Medicine, New York, NY, USA; 21 Clinical & Translational Science Center, Weill Cornell Medicine, New York, NY, USA; 22 Department of Health Outcomes and Biomedical Informatics, University of Florida, Gainesville, FL, USA; 23 Department of Neurology, University of Pittsburgh, Pittsburgh, PA, USA; 24 Scripps Research, Scripps Research Translational Institute, La Jolla, CA, USA

**Keywords:** Translational research, electronic health records, natural language processing, network, ENACT

## Abstract

**Objective:**

Electronic Health Record (EHR) data are critical for advancing translational research and AI technologies. The ENACT network offers access to structured EHR data across 57 CTSA hubs. However, substantial information is contained in clinical narratives, requiring natural language processing (NLP) for research. The ENACT NLP Working Group was formed to make NLP-derived clinical information accessible and queryable across the network.

**Methods:**

We established the ENACT NLP Working Group with 13 sites selected based on criteria including clinical notes access, IT infrastructure, NLP expertise, and institutional support. We divided sites into five focus groups targeting clinical tasks within disease contexts. Each focus group consisted of two development sites and two validation sites. We extended the ENACT ontology to standardize NLP-derived data and conducted multisite evaluations using the Open Health Natural Language Processing (OHNLP) Toolkit.

**Results:**

The working group achieved 100% site retention and deployed NLP infrastructure across all sites. We developed and validated NLP algorithms for rare disease phenotyping, social determinants of health, opioid use disorder, sleep phenotyping, and delirium phenotyping. Performance varied across sites (F1 scores 0.53–0.96), highlighting data heterogeneity impacts. We extended the ENACT common data model and ontology to incorporate NLP-derived data while maintaining Shared Health Research Informatics NEtwork (SHRINE) compatibility.

**Conclusion:**

This demonstrates feasibility of deploying NLP infrastructure across large, federated networks. The focus group approach proved more practical than general-purpose approaches. Key lessons include the challenge of data heterogeneity and importance of collaborative governance. This work also provides a foundation that other networks can build on to implement NLP capabilities for translational research.

## Introduction

Electronic health record (EHR) data serve as a rich and invaluable source of real-world clinical information, enabling researchers and healthcare professionals to gain comprehensive insights into patient populations, treatment outcomes, and healthcare practices [1]. By capturing a broad spectrum of clinical information, including demographic details, diagnosis, procedures, medications, laboratory test results, and clinical notes, EHR systems create a longitudinal record that mirrors the complexity and heterogeneity of modern healthcare. The accessibility of EHR data is paramount for advancing translational research and the application of cutting-edge technologies, including artificial intelligence and machine learning. Furthermore, these computational tools depend on robust, standardized, and interoperable EHR datasets to enable predictive modeling [2], automated digital phenotyping [3], risk stratification [4], and clinical decision support systems [5] that can enhance clinical effectiveness, improve patient safety [5,6], and ultimately shape the future of healthcare delivery.

The national Evolve to Next-Gen Accrual to Clinical Trials (ENACT) network [1] was established in 2015 as the Accrual to Clinical Trials (ACT) network to enable cohort discovery from EHR data. This federated network connects EHR data repositories across 57 Clinical and Translational Science Awards (CTSA) hubs, enabling researchers to query the data of more than 142 million patients across the hubs (sites). The ENACT network integrates local Informatics for Integrating Biology at the Bedside (i2b2) [7] and Observational Medical Outcomes Partnership (OMOP) [8] data repositories (and eventually PCORnet [9] data repositories) through the Shared Health Research Information Network (SHRINE) platform, which enables interactive querying of the data [10]. The network’s data, encompassing structured EHR information on demographics, diagnoses, procedures, medications, laboratory test results, and visits, extends back at least a decade, with some sites providing data for up to two decades. Updates to the data occur at least once a month.

The ACT network aimed to enable national cohort discovery, particularly for multisite research such as clinical trials, including those supported by the Trial Innovation Network (TIN) [11]. However, ENACT“s goal is broader, including large-scale clinical and translational research using patient counts, distributed analytics, and ephemeral analytics enclaves. Furthermore, ENACT provides prep-to-research data, enables the generation of evidence for clinical decision-making, and serves as a resource for educating trainees.

Currently, ENACT provides access to structured EHR data, enabling significant advances in cohort discovery and research across this national network. However, while structured EHR data offers valuable insights, much clinical information remains embedded within unstructured EHR data. The unstructured EHR data, including clinical encounter notes, radiology reports, pathology reports, and other narrative documents, are challenging to analyze due to their free-text format. To harness the full potential of EHRs for translational research, applying natural language processing (NLP) to extract research-usable data from clinical notes is essential. Recognizing this critical need, the ENACT NLP Working Group was established to make NLP-derived data accessible and queryable across the network. Such data will enhance the analytical capacity of the network, enabling researchers to tap into previously inaccessible information in clinical notes and generate new insights that can drive advances in translational research and clinical care.

This article provides a comprehensive overview of the development and deployment of NLP infrastructure in ENACT. We describe the formation and goals of the working group, the policies and logistics involved, and the specific NLP algorithms and tools utilized. We also describe the extension of the ENACT ontology to standardize and query NLP-derived data across the network. Furthermore, we provide a practical guide on multisite evaluation of NLP algorithms, highlighting their performance, scalability, and adaptability across diverse healthcare systems. We also include an in-depth reflection on the experiences and lessons learned from this journey, which may be helpful in other national data networks, such as the PCORnet [9] and the All of Us Research Program [12], which use NLP to unlock the potential of clinical notes for research.

## Methods

### Formation and organization of the ENACT NLP working group

We established the ENACT NLP Working Group in 2023 with participation from 13 ENACT sites selected based on specific technical and organizational criteria. Eligible sites were required to have: (1) an accessible source of clinical notes (e.g., clinical data warehouse or Epic Clarity reporting database), (2) existing information technology infrastructure capable of supporting NLP computation, (3) demonstrated NLP expertise among staff, and (4) institutional support from local CTSA hub leadership for obtaining clinical notes access and managing participation logistics.

The recruitment process began with a comprehensive survey of all 57 ENACT sites to assess technical capabilities, resource availability, and institutional interest. Sites meeting the primary eligibility criteria were invited to participate in detailed technical assessments, including infrastructure readiness evaluations and personnel capability reviews. The final site selection prioritized geographic diversity, healthcare system variety (including academic medical centers, integrated health systems, and specialty hospitals), and complementary technical expertise to ensure robust representation across the federated network.

### NLP implementation strategy evaluation

We systematically evaluated potential implementation strategies for federated clinical NLP deployment. This evaluation process assessed multiple approaches, including general-purpose clinical NLP platforms versus specialized, domain-specific algorithms. The assessment criteria included: (1) compatibility with ENACT’s existing SHRINE infrastructure and common data model (CDM) implementations, (2) preprocessing requirements based on site-specific data characteristics, (3) resource allocation efficiency, and (4) alignment with CTSA research requirements.

### Focus group strategy and resource optimization

We divide participating sites into five specialized focus groups, each targeting specific clinical tasks within well-defined disease or condition contexts. This specialization approach is based on three key principles: (1) Clinical Relevance: Each focus group addresses clinically important research questions with clear translational implications; (2) Technical Feasibility: Focus group tasks are scoped to be achievable within available resources while maintaining high technical standards; and (3) Resource Efficiency: Focus group organization minimizes duplication of effort by leveraging existing funded projects and ongoing research initiatives at participating sites.

Each focus group is structured with two development sites responsible for collaborative algorithm design, initial validation, and comprehensive documentation, and at least two additional validation sites responsible for independent cross-site evaluation and generalizability assessment. This structure balances the need for intensive development effort with robust cross-site validation while maintaining manageable coordination complexity.

### Working group governance and communication framework

We designed a collaborative governance model to coordinate complex multi-institutional activities while respecting institutional autonomy and diverse organizational cultures. The governance framework emphasizes shared decision-making, transparent communication, and equitable resource allocation across participating sites.

The working group operates under a distributed leadership model with rotating meeting facilitation and consensus-based decision-making protocols. Leadership responsibilities are shared among sites based on expertise areas, technical decisions are made through working group consensus, and administrative coordination is managed through a dedicated project management personnel. This structure ensures that no single institution dominates decision-making while maintaining efficient coordination across diverse institutional environments.

## Results

### The ENACT NLP Working Group

**Figure 1.**
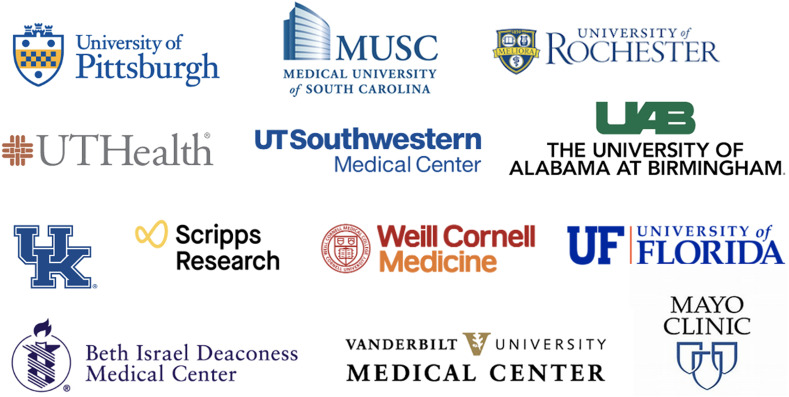
Participating sites in the evolve to next-gen accrual to clinical trials (ENACT) network natural language processing (NLP) working group.

The ENACT NLP Working Group successfully launched with 13 participating sites representing diverse healthcare systems across the United States (See Figure 1). All participating sites met the established criteria and successfully established the required technical infrastructure, including access to clinical notes, computational resources, and previous experience in clinical NLP. The working group achieved 100% site retention throughout the project period, thanks to effective coordination facilitated by the established communication framework.

During the initial discussions, we discovered two challenges: (1) Processing all clinical notes at each site (which could number in the billions) and extracting every biomedical entity from each note (which could number in the tens of thousands) was infeasible, and (2) Deploying a general-purpose NLP algorithm capable of extracting all entities proved unrealistic and unlikely to perform optimally. Instead, specialized NLP algorithms targeting specific entities for particular tasks with greater precision were deemed more realistic and practical.

Further, to maximize the limited funding available to the working group, we divided the participating sites into five focus groups, each targeting a specific task in the context of a disease or condition. Each focus group consisted of two development sites and at least two additional validation sites. The development sites were tasked with jointly designing and validating a specialized NLP algorithm, while the validation sites were responsible for evaluating the algorithm. After validation, the NLP algorithm can be deployed across the entire network. Some development sites leveraged already-funded local projects or focused on an algorithm already in development for an ongoing project. This strategy significantly reduced the resources and effort needed to develop and deploy several algorithms. Table 1 lists the focus groups, associated development sites, and validation sites.

**Table 1.** Focus group tasks and associated development sites, deployment sites, cohort definitions, and clinical note types

Focus group task	Development sites	Validation sites	Cohort definition	Note type(s)
Rare disease phenotyping	University of Texas Health Science Center at Houston, Mayo Clinic	University of Pittsburgh, Weill Cornell Medicine	Patients with any of the following conditions: Complex Regional Pain Syndrome (CRPS), Trigeminal Neuralgia (TN), Idiopathic Pulmonary Fibrosis (IPF), Familial Pancreatic Carcinoma (FPC),, and Primary sclerosing cholangitis (PSC) identified by ICD–10 codes (G90.5*, G50.0, J84.112, C25.*, K83.01).	Any
Social determinants of health (SDOH) – Housing status	University of Kentucky, University of Pittsburgh	University of Rochester, University of Texas Health Science Center at Houston	Patients with at least one Emergency Department visit (see Harris et al. [13] for details)	Clinical notes for ED visits (e.g., comprehensive assessment, consultation, etc.)
Opioid use disorder	Medical University of South Carolina, University of Kentucky	University of Pittsburgh, University of Rochester	Patients with any of the following conditions: Opioid Overdose (see Lenert et al. [14] and Ward et al. [15] for details), Opioid Use Disorder (see Zhu et al. [16] for details)	Emergency Department notes
Sleep phenotyping	University of Pittsburgh, University of Florida	University of Texas Health Science Center at Houston, University of Rochester	Patients with Alzheimer’s disease (see Venkatesh et al. [17] for details)	Clinical encounter notes
Delirium phenotyping	Mayo Clinic and Olmsted Medical Center	University of Texas Health Science Center at Houston, University of Pittsburgh, Vanderbilt University Medical Center, Beth Israel Deaconess Medical Center, University of Texas Southwestern Medical Center, University of Alabama at Birmingham	Patients with delirium (see St Sauver et al. [18] for details)	Clinical notes (primarily focus on progress, nursing, and consultation notes (e.g., neurology/psychiatry consultations, physical/occupational therapy, social work))

During later discussions, we realized that designing a specialized NLP algorithm targeted for a task required specifying the*patient cohort*and the*clinical note type*. For example, for the SDOH–Housing Status task, the patient cohort included individuals with substance use disorder (specifically those with stimulant and opioid use disorders (OUDs) with ICD-10-CM diagnosis codes of F11.*, F14.*, F15.*, T40.*, and T43.6*), and EHR data was limited to emergency department notes. The algorithm would not be expected to be applied to other types of patients or notes. Thus, we required each focus group to provide a clear cohort definition and identify the type of note needed to develop the NLP algorithm. Table 1 provides the cohort definitions and clinical note types identified by each focus group.

### NLP implementation strategies considered

Our systematic evaluation of potential implementation strategies for federated clinical NLP deployment revealed critical insights that shaped our strategic approach. We assessed multiple approaches, including general-purpose platforms that extract standardized medical concepts versus specialized, domain-specific algorithms. A standardized strategy would have processed clinical notes to extract broad medical concepts, such as Unified Medical Language System (UMLS) concepts, and populated existing observation_fact tables within the ENACT CDM, enabling researchers to query NLP-derived concepts through the established SHRINE interface. The Open Health Natural Language Processing (OHNLP) Consortium’s Toolkit, with its exceptional flexibility in processing diverse data formats, compatibility with both i2b2 and OMOP data models, and adaptability to site-specific variations, positioned it as the optimal solution for handling ENACT’s heterogeneous data environments. Given the universal need for substantial preprocessing infrastructure and CTSA investigators’ requirements for fine-grained clinical phenotypes, we determined that the specialized focus group approach using OHNLP’s adaptable framework would provide superior precision for targeted clinical applications while optimizing limited resources within ENACT’s established federated architecture. During this process, the NLP working group has worked collaboratively on selecting and implementing NLP tools for extracting medical concepts from clinical notes. The team also extended the ENACT CDM to incorporate NLP-derived data while maintaining flexibility for different projects. Standardized conventions for storing NLP-extracted entities and contextual attributes were established, ensuring seamless integration with structured EHR data. Additionally, extensions to the ENACT ontology were developed to facilitate querying NLP-derived concepts across ENACT, and a federated evaluation framework was introduced for cross-site validation of NLP algorithms. To enhance reliability, the team also implemented a standardized error analysis process, utilizing an established taxonomy to refine model performance and assess generalizability across institutions. These collective efforts streamlined clinical textual analytical capabilities within ENACT. Details about these technologies are in the Supplemental Material.

### Overview of the ENACT NLP workflow

**Figure 2.**
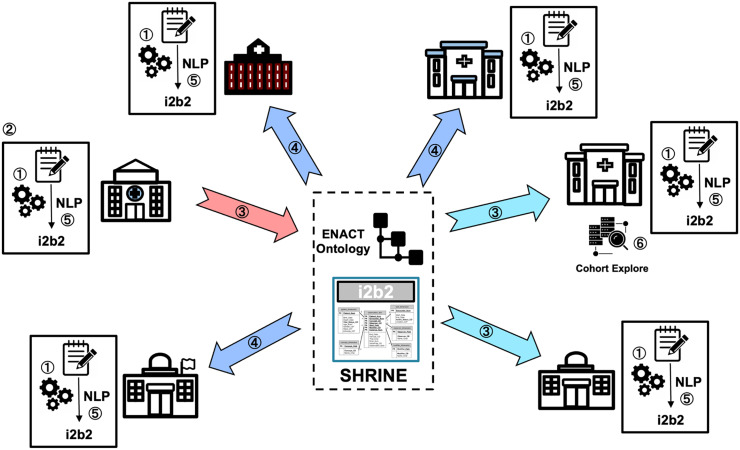
An overview of the ENACT NLP workflow. *SHRIN= shared health research information network.

Figure 2 presents an overview of the NLP workflow developed by the ENACT NLP Working Group, which is described step by step below.

① NLP Infrastructure: Each site participating in the NLP initiative identifies a source of clinical notes (such as a clinical data warehouse or Epic’s Clarity), allocates computing resources for NLP processing, and deploys the OHNLP Toolkit. In addition to ENACT’s Institutional Review Board (IRB) approval for structured EHR data, the site obtains additional IRB approval, if necessary, to use clinical notes.

② Algorithm Development: A participating site proposes and develops an NLP algorithm, contacts the working group to coordinate its validation, and partners with the Data Harmonization Working Group to create the ENACT ontology extension necessary for deploying the NLP algorithm.

③ Algorithm Validation: The site that developed the algorithm shares it with validation sites, along with the associated specifications, such as the cohort definition, note type, and process for establishing the gold and silver reference standards, as directed by the working group.

④ Dissemination: Following validation, the working group disseminates the algorithm and associated specifications to the network via an online repository such as GitHub. The working group also coordinates with the Data Harmonization Working Group and the Network Operations Working Group to deploy the ontology extensions across the network.

⑤ Site Integration: Each participating site downloads the algorithm and associated specifications from the online repository, integrates it into their local NLP infrastructure, and populates the local ENACT data repository’s i2b2 observation_fact table with NLP-derived data. The site also updates the ENACT ontology to include the extension required for querying NLP-derived data.

⑥ Researcher Use: Any ENACT researcher at any participating site uses the SHRINE interface to create queries that search NLP-derived and structured EHR data. The query returns patient counts from participating sites in the same manner as existing structured EHR data queries.

This workflow, which includes infrastructure needs, algorithmic creation and validation, network-wide distribution, and local integration, provides a structured approach for introducing and scaling NLP capability in the network.

### Demonstration projects

In this section, we present four demonstration projects, each representing the work of a focus group and showcasing a distinct area of research. Each project is at a different stage of development, reflecting variations in goals, challenges, and resource availability. While some focus groups have made significant progress and are close to integrating NLP-derived data into the network, others are in the early stages, concentrating on foundational tasks such as acquiring clinical notes, establishing NLP infrastructure, or refining the NLP algorithm. This variation underscores the dynamic and adaptive nature of the collaborative effort to develop and disseminate NLP capabilities across a large national network.

### Demonstration project 1: Sleep phenotyping focus group

The Sleep Phenotyping Focus Group is investigating sleep phenotyping within a cohort of Alzheimer’s Disease (AD) patients, using encounter notes in these patients. To extract relevant sleep phenotype information, we previously developed an NLP algorithm to extract key phenotypes such as snoring, napping, sleep problems, poor sleep quality, daytime sleepiness, nocturnal awakenings, sleep duration, and other nocturnal symptoms [19]. This project offers a structured approach for analyzing the sleep disturbances commonly observed in AD patients, ultimately contributing to a deeper understanding of their clinical implications.

The evaluation framework described earlier was applied to assess the NLP algorithm, and the performance is shown in Table 2. Two sites, University of Pittsburgh (Pitt) and the University of Florida (UF) are the development sites, while the University of Texas Health Science Center at Houston (UTH) is one of the validation sites (the additional validation site, the University of Rochester (UR), is in the process of generating validation results). The algorithm’s behavior varied among the three sites. At Pitt, it had a high recall and low precision, indicating that it functioned with high sensitivity, whereas at UF and UTH, it had a low recall and high precision, indicating that it functioned more cautiously. Part of the reason for this discrepancy is that the data at Pitt is more evenly distributed across the sleep phenotypes, with sample sizes of 12 and 48 for 6 of the 9 concepts, whereas the data at UTH and UF is unevenly distributed mainly for sleep problems and sleep quality concepts, for which the algorithm generates many false negatives.

**Table 2.** Performance of the algorithm developed by the sleep phenotyping focus group

	ENACT sites		
Metrics	Pitt	UF	UTH
F1	0.776	0.647	0.698
Recall	0.944	0.542	0.551
Precision	0.695	0.868	0.953

### Demonstration project 2: Social determinants of health – housing status focus group

Housing is a key environmental social determinant of health (SDOH), closely associated with mortality and clinical outcomes. Housing Status Focus Group seeks to develop an NLP algorithm to extract the housing status of individuals from emergency department notes. This project aims to provide valuable insights into the impact of housing instability on health outcomes, thereby informing future interventions and support strategies.

The housing status NLP algorithm was developed to extract housing-related concepts such as homelessness, unstable housing, recovery housing, emergency housing, temporary housing, and exposure, and its performance is shown in Table 3. The University of Kentucky (UK) and Pitt are the development sites, and UR and UTH are the validation sites. Each site created a gold standard for evaluation using a specific subset of patients treated in emergency departments; details of the NLP algorithm development and evaluation can be found in the relevant publication [13].

**Table 3.** Performance of the algorithm developed by the housing status focus group

	ENACT sites			
Metrics	UK	Pitt	UR	UTH
F1	N/A	0.959	0.823	0.689
Recall	0.980	0.992	0.798	0.875
Precision	0.990	0.928	0.850	0.568

### Demonstration project 3: Opioid use disorder focus group

Patients who present to the Emergency Department with an opioid overdose (OOD) are at significant risk of death [20]. Identifying individuals with OUD and at risk of OOD can aid in better treatment and counseling, particularly in the context of treating acute and chronic pain with opioids. ICD codes can identify OUD patients and those at risk of OOD, but they may not be available in the EHR at the time of the visit (Ward et al. [15]), and they are frequently absent in patients when evidence in their unstructured clinical notes indicates a risk for OUD in Zhu et al. [16] . In particular, Zhu et al. [16] found that a lexicon-based strategy for identifying patients at risk for OUD outperformed an ICD-based method for phenotyping patients with OUD. The OUD Focus Group is formalizing a phenotype based on the ICD code approach (Ward et al. [15], Lenert et al. [14] and Zhu et al. [16]) to be used as an initial silver standard for evaluation. The initial NLP phenotyping method will be based on Zhu et al.’s lexicon-based approach to identifying OUD, which will be refactored to work in the OHNLP framework. The NLP algorithm is currently being developed and validated across multiple sites.

### Demonstration project 4: Delirium phenotyping focus group

Delirium is a common geriatric syndrome characterized by an acute change in mental status, fluctuating course, lack of attention, and disorganized thinking or altered level of consciousness [21]. Accurate prediction of delirium could significantly improve patient outcomes through targeted interventions for hospitalized patients. For delirium case ascertainment, we used the Confusion Assessment Method (CAM) [22], which is recommended by the Network for Investigation of Delirium: Unifying Scientists (NIDUS), as the gold standard for diagnosing delirium. NLP-CAM is an NLP-powered computational phenotyping tool that can identify a patient’s delirium status from the EHR [23]. The tool was initially developed at the Mayo Clinic (Mayo) based on CAM and includes 13 unique concepts that range from neuropsychological characteristics to cognitive and memory problems (e.g., agitation, disorganized thinking, and fluctuation). We applied NLP-CAM to three test sites (UTH, University of Alabama at Birmingham (UAB), and Vanderbilt University Medical Center (VUMC)) and reported the out-of-the-box performance, as shown in Table 4. We observed moderate to high performance degradation due to site variations in CAM screening, documentation, and patient characteristics. Our next step is to conduct federated refinement [24] to optimize NLP performance at each site.

**Table 4.** Performance of the algorithm developed by the delirium phenotyping focus group

	ENACT sites			
Metrics	Mayo	UTH	UAB	VUMC
F1	0.958	0.895	0.530	0.606
Recall	0.919	0.985	0.770	0.796
Precision	1.000	0.819	0.400	0.490

## Discussion

The ENACT NLP Working Group created NLP capability that is specifically suited to a multisite, federated network for supporting large-scale analytics. This capability enables extracting data from clinical narratives, facilitating research that involves collaboration across multiple sites, while simultaneously addressing data heterogeneity and scalability. Below, we highlight six areas where the NLP capability offers transformative potential in ENACT.

### Potential applications

#### Recruitment for Clinical trials

NLP-derived data combined with structured EHR data could enhance participant identification for multisite clinical trials, improving recruitment efficiency and demographic representation. This capability is particularly valuable for rare disease research, where pooling data across institutions enables the identification of sufficient cases for robust analysis.

#### Multisite large cohort studies

The integration of unstructured data into large cohort studies significantly enhances the granularity and scope of research. ENACT NLP enables the identification of complex phenotypes, such as those described in clinical narratives, which are often omitted in structured data alone. This capability is particularly valuable for studying rare diseases, as it allows researchers to pool data from multiple institutions to identify sufficient cases for robust analysis. By supporting large-scale phenotyping and longitudinal analyses, ENACT NLP facilitates cohort studies that can uncover complex relationships between clinical variables and outcomes, providing insights that would be limited with data from a single site.

#### Federated learning and artificial intelligence (AI) development

The infrastructure supports privacy-preserving AI model development by enabling local processing of unstructured data with centralized model aggregation. This approach addresses data heterogeneity and privacy challenges while creating models generalizable across diverse healthcare systems.

#### Digital twin

Digital twins – virtual patient representations that simulate disease progression and treatment responses – benefit from ENACT NLP’s multisite capabilities. By extracting nuanced patient data from unstructured narratives across the network, ENACT NLP enables the creation of comprehensive digital twins that capture diverse clinical contexts and patient populations. These models support personalized simulations for precision medicine applications at scale.

#### Population health and surveillance

ENACT NLP supports epidemiological studies and real-time public health surveillance by extracting disease patterns, healthcare utilization metrics, and social determinants of health from clinical narratives. These capabilities enable targeted interventions and evidence-based policy decisions.

#### Clinical decision support

The multisite network enhances clinical decision-making by enabling clinicians to identify similar patients across institutions and review their treatment outcomes. This is particularly impactful for rare or complex conditions where local data may be insufficient.

#### Additional applications

ENACT NLP’s multisite infrastructure supports diverse research areas: precision medicine (extracting patient-specific genetic and environmental factors for personalized care); quality improvement (identifying workflow inefficiencies and care gaps from clinical narratives); health equity research (analyzing social determinants like housing instability and food insecurity); pharmacovigilance (detecting adverse drug reactions and off-label usage patterns); and healthcare education (providing real-world case studies for training programs). These applications collectively expand ENACT NLP’s impact across healthcare research, policy, and practice.

#### Lessons learned

Implementing and deploying NLP infrastructure in ENACT has been a multifaceted journey, marked by significant advancements in integrating NLP and textual analytical capabilities into a large national EHR network. The ENACT NLP Working Group’s collaborative efforts, leveraging existing IT infrastructures and NLP expertise at various CTSA hubs, facilitated the rapid deployment of NLP pipelines across the network. Establishing dedicated communication channels through Slack workspaces and regular coordination meetings proved instrumental in ensuring smooth coordination and troubleshooting among participating sites.

#### Data heterogeneity: the fundamental implementation challenge

Our most critical lesson learned was that data heterogeneity across sites, even within sites using the same EHR vendor, represents the fundamental implementation challenge. Despite 9 of 13 sites using Epic as their primary EHR system, we encountered substantial heterogeneity in their note data. Each site utilized different data access sources (clinical data warehouse, Epic Clarity, or OMOP warehouse) with unique challenges: template variations across departments and time periods, loss of formatting during extract, transform, load (ETL) processes, character encoding issues, inconsistent use of structured fields, and site-specific documentation workflows.

Sites demonstrated striking diversity in how clinical information was structured and stored. Pitt’s dual Epic-Cerner environment required processing both mixed vendor templates and legacy data integration challenges. The Medical University of South Carolina (MUSC) faced critical ETL issues where structurally significant formatting, including tables and newlines, was stripped during data warehouse transfer, while UAB’s Cerner system complicated matters by converting notes to portable document format (PDF) files. Character encoding problems plagued multiple sites, with MUSC encountering Windows-1250 encoding flagged as ASCII/Unicode and Mayo experiencing Unicode-related NLP failures.

Structured assessment templates were unexpectedly exported as unstructured text blocks across multiple sites, including UT Southwestern’s risk screening templates, UTH’s nursing flow sheets, UK’s nursing assessments, Mayo’s medication code. Pitt’s Epic system created additional complications by automatically duplicating notes after physician signatures. These issues required specialized parsers for text extraction, duplicate detection, and data reconciliation, extending implementation timelines by 3–4 weeks.

#### Broader implementation challenges

Beyond data heterogeneity, there were several other implementation challenges. The sheer volume of unstructured clinical notes made processing all data impractical, forcing us to prioritize specific concepts rather than attempt comprehensive extraction of all concepts. Limited funding further restricted the number of NLP algorithms we could develop and evaluate. Implementation timelines varied significantly due to differences in site expertise, resource availability, and IRB approval delays, with some sites first validating on synthetic data before transitioning to gold-standard datasets.

The domain-specific algorithm approach demands significant infrastructure investment. Each site must maintain: (1) computing resources capable of processing millions of notes (minimum 16 cores, 64 GB RAM for production), (2) secure storage for raw and NLP-derived data, (3) at least 0.5 full-time equivalent (FTE) technical personnel with NLP expertise for customization and maintenance, and (4) sustained funding for updates and validation. These requirements, particularly specialized technical expertise and computational resources, may exclude smaller institutions or those with limited informatics infrastructure from participation.

#### Future directions

For the implementation and deployment of NLP in ENACT, we did not use a theoretical framework to guide the implementation process. Implementation science offers many valuable frameworks, such as the Exploration, Preparation, Implementation, Sustainment Framework (EPIS) [25,26] and the Consolidated Framework for Implementation Research (CFIR) [25]. These frameworks offer systematic methods to address site-specific adaptations, optimize workflows, and identify scalability barriers, potentially accelerating the translation of NLP insights into clinical practice across diverse healthcare environments.

## Limitations

The process described in this article has several limitations that warrant consideration. First, the focus on specific projects, while necessary due to resource constraints, limits generalizability to other clinical contexts. NLP models trained and validated on specific datasets may not perform as effectively on different patient populations, specialties, or healthcare settings, requiring additional adaptation and validation efforts. Additionally, the variability in data quality, note types, and EHR systems across the participating sites poses challenges in ensuring consistent performance of the NLP algorithms. Differences in documentation practices, clinical terminologies, and system configurations could introduce inconsistencies that affect model robustness and accuracy, necessitating site-specific tuning. The reliance on local funding and existing funded projects for developing specific NLP tools also introduces potential biases, as the algorithms may be optimized for specific datasets not representative of the broader population. This funding-driven approach may inadvertently prioritize projects with greater institutional support while leaving gaps in NLP capabilities for underrepresented patient groups and clinical domains.

Second, the network-wide querying function remains under development, limiting immediate utility for large-scale multisite research. While some sites have joined the ENACT test network to refine querying capabilities, progress has been gradual due to infrastructure complexity and personnel bandwidth constraints. We anticipate that querying functionality will be available to working group sites by late 2025 and network-wide by late 2026.

Third, we did not systematically evaluate alternative NLP infrastructure solutions. For example, Apache cTAKES offers built-in stripping of protected health information and NLM Metathesaurus integration that might have benefited certain use cases. While the OHNLP Toolkit proved adequate for our approach, a comprehensive comparison of available infrastructures, including commercial or cloud-based solutions, might have revealed alternative solutions to challenges like cross-site portability or maintenance requirements.

### Generative AI and LLMs in ENACT NLP

Recent advances in generative AI (GenAI) and large language models (LLMs) have the potential to address several key limitations in the ENACT NLP project. First, current limitations in developing generalized NLP algorithms across diverse health systems could be alleviated using LLMs. Unlike specialized NLP algorithms, LLMs such as GPT-4 can be fine-tuned to understand clinical context across various datasets without requiring domain-specific rules. This generalization ability could help ENACT develop more versatile NLP tools to handle multiple clinical tasks (e.g., phenotyping, cohort identification) across different sites without extensive retraining. Second, LLMs could enhance the accuracy of phenotyping efforts, particularly in multisite studies, where heterogeneity in data sources makes consistent concept extraction difficult. GenAI models, particularly those trained on clinical data, can significantly enhance this task by capturing the nuances of clinical language. LLMs can interpret complex medical narratives more effectively than rule-based systems and adapt to new or evolving medical terminologies [27]. For example, open-source LLMs (e.g., Llama2-70B-chat, Openchat-3.5-0106) could identify mammograms that required follow-up with F1 = 1.0 (i.e., perfect performance in that experiment) based on text reports. Notably, mammography reports include a Breast Imaging-Reporting and Data System (BI-RADS) score. A mammogram (report) that requires follow-up is one where the interpreting radiologist assigned a BI-RADS score other than 1 or 2. Thus, identifying mammograms that require follow-up is a relatively simple information extraction task [28]. Third, LLMs may reduce the time to develop NLP algorithms. LLMs offer the advantage of being pre-trained on diverse datasets, enabling them to incorporate time-consuming external knowledge into knowledge engineering in traditional rule-based NLP systems. Fourth, LLMs could automate multisite validation and deployment. One of the key bottlenecks for ENACT is the complex logistics of multisite validation of NLP tools. GenAI models could streamline this by providing automated validation.

While GenAI and LLMs offer considerable potential to advance the ENACT NLP initiative, several significant challenges and drawbacks must be considered. First, data privacy and security concerns are paramount in healthcare, as LLMs typically require large amounts of data to train and fine-tune. This presents the risk of inadvertently exposing sensitive patient information, especially when models are trained across multiple sites in a distributed network like ENACT. Even anonymized or de-identified data may still contain subtle details that could re-identify individuals, posing a significant risk under regulations such as HIPAA. Additionally, the computational and resource costs associated with training, fine-tuning, and deploying LLMs are substantial, and for a large, multisite initiative like ENACT, these infrastructure costs could be prohibitive for sites with limited resources, leading to disparities in model performance and inconsistent results across the network. Another concern is fairness, as LLMs often inherit biases from their training data. In healthcare, biased models could disproportionately affect certain demographic groups, leading to incorrect or harmful clinical recommendations. This is particularly problematic in NLP tasks such as phenotyping or clinical decision support, where subtle language or data representation biases could skew interpretations. The black box nature of LLMs also poses challenges in clinical applications where interpretability is crucial, as clinicians and researchers often need to understand why a model made a particular prediction. This lack of explainability can lead to a lack of trust in the healthcare domain. Using LLMs raises numerous ethical and legal concerns, including patient consent, data ownership, and responsibility for errors or adverse outcomes. These issues are particularly complex in a multisite network like ENACT, where multiple stakeholders may be involved in data sharing, algorithm development, and model deployment. Moreover, LLMs sometimes generate plausible but incorrect information, a phenomenon known as hallucination. This could have serious consequences in clinical contexts, leading to misinformed decisions based on faulty data extraction, summarization, or interpretation of clinical narratives. While GenAI and LLMs hold promises for advancing the work of ENACT NLP, the challenges in their implementation, especially in areas like privacy, bias, and explainability, must be carefully managed. A balanced approach combining the power of LLMs with traditional rule-based methods and rigorous oversight may offer the best path forward for ENACT NLP objectives.

## Conclusion

The ENACT NLP Working Group has made significant strides in deploying NLP infrastructure across a large, federated data network, leveraging existing IT infrastructure and NLP expertise from several CTSA hubs. By establishing focus groups dedicated to specific disease conditions and utilizing the OHNLP Toolkit, the working group was able to target specialized NLP algorithms for distinct clinical tasks. This pragmatic approach has enabled ENACT to deploy NLP solutions more efficiently while addressing each site’s unique data and resource challenges. Furthermore, the collaborative framework of partnerships within the OHNLP development team has been crucial in facilitating rapid implementation and troubleshooting.

The project has faced challenges in processing vast amounts of clinical notes and developing NLP algorithms that perform consistently across all sites. The working group opted for focused NLP deployments and clearly defined cohort specifications to address these obstacles, tailoring algorithms to specific note types and clinical contexts. As the project evolves, creating and refining these NLP tools while emphasizing collaboration and resource sharing will be critical in broadening the scope and impact of the ENACT NLP initiative across diverse healthcare environments.

## Supporting information

10.1017/cts.2025.10116.sm001 Wang et al. supplementary material Wang et al. supplementary material

## Data Availability

The patient-level electronic health record data cannot be shared due to privacy and legal concerns. The software utilized in this study is primarily open source. The OHNLP Toolkit is available at the OHNLP website (https://ohnlp.org/). The ENACT ontology can be accessed at ENACT Network Resources (https://www.enact-network.us/resources/technical). Documentation for the ENACT CDM is available at the i2b2 Wiki (https://community.i2b2.org/wiki/display/BUN/i2b2+Common+Data+Model+Documentation).

## Supplementary material

The supplementary material for this article can be found at https://doi.org/10.1017/cts.2025.10116

## References

[1] Visweswaran S , Becich MJ , D’Itri VS , et al. Accrual to clinical trials (ACT): A Clinical and Translational Science Award Consortium Network. JAMIA Open 2018;1:147–152. 30474072 10.1093/jamiaopen/ooy033 PMC6241502

[2] Tang AS , Woldemariam SR , Miramontes S , Norgeot B , Oskotsky TT , Sirota M. Harnessing EHR data for health research. Nat Med 2024;30:1847–1855. 38965433 10.1038/s41591-024-03074-8

[3] Zhang Y , Cai T , Yu S , et al. High-throughput phenotyping with electronic medical record data using a common semi-supervised approach (PheCAP). Nat Protoc 2019;14:3426–3444. 31748751 10.1038/s41596-019-0227-6 PMC7323894

[4] Xu D , Wang C , Khan A , et al. Quantitative disease risk scores from EHR with applications to clinical risk stratification and genetic studies. NPJ Digit Med 2021;4:116. 34302027 10.1038/s41746-021-00488-3 PMC8302667

[5] Sutton RT , Pincock D , Baumgart DC , Sadowski DC , Fedorak RN , Kroeker KI. An overview of clinical decision support systems: Benefits, risks, and strategies for success. NPJ Digit Med 2020;3:17. 32047862 10.1038/s41746-020-0221-y PMC7005290

[6] Meeks DW , Smith MW , Taylor L , Sittig DF , Scott JM , Singh H. An analysis of electronic health record-related patient safety concerns. J Am Med Inform Assoc 2014;21:1053–1059. 24951796 10.1136/amiajnl-2013-002578 PMC4215044

[7] Murphy SN , Weber G , Mendis M , et al. Serving the enterprise and beyond with informatics for integrating biology and the bedside (i2b2). J Am Med Inform Assoc 2010;17:124–130. 20190053 10.1136/jamia.2009.000893 PMC3000779

[8] Hripcsak G , Duke JD , Shah NH , et al. Observational health data sciences and informatics (OHDSI): Opportunities for observational researchers. Stud Health Technol Inform 2015;216:574–578. 26262116 PMC4815923

[9] Fleurence RL , Curtis LH , Califf RM , Platt R , Selby JV , Brown JS. Launching PCORnet, a national patient-centered clinical research network. J Am Med Inform Assoc 2014;21:578–582. 24821743 10.1136/amiajnl-2014-002747 PMC4078292

[10] McMurry AJ , Murphy SN , MacFadden D , et al. SHRINE: Enabling nationally scalable multi-site disease studies. PloS One 2013;8:e55811. 23533569 10.1371/journal.pone.0055811 PMC3591385

[11] Bernard GR , Harris PA , Pulley JM , et al. A collaborative, academic approach to optimizing the national clinical research infrastructure: The first year of the Trial Innovation Network. J Clin Transl Sci 2018;2:187–192. 31011433 10.1017/cts.2018.319 PMC6474372

[12] All of Us Research Program Investigators, Denny JC , Rutter JL , et al. The “All of Us Research program. N Engl J Med 2019;381:668–676. 31412182 10.1056/NEJMsr1809937 PMC8291101

[13] Harris DR , Fu S , Wen A , et al. The ENACT network is acting on housing instability and the unhoused using the open health natural language processing toolkit, Journal of Clinical and Translational Science. Cambridge University Press, 2024:8, e98. 39655040 10.1017/cts.2024.543 PMC11626605

[14] Lenert LA , Zhu V , Jennings L , et al. Enhancing research data infrastructure to address the opioid epidemic: The Opioid Overdose Network (O2-Net). JAMIA Open 2022;5:ooac055. 35783072 10.1093/jamiaopen/ooac055 PMC9243402

[15] Ward R , Obeid JS , Jennings L , et al. Enhanced phenotypes for identifying opioid overdose in emergency department visit electronic health record data. JAMIA Open 2023;6:ooad081. 38486917 10.1093/jamiaopen/ooad081 PMC10938047

[16] Zhu VJ , et al. Automatically identifying opioid use disorder in non-cancer patients on chronic opioid therapy. Health Inform J 2022;28:14604582221107808. 10.1177/14604582221107808 PMC10826411 35726687

[17] Venkatesh S , Wang L , Cai T , Xia Z. A real-world investigation of demographic disparity in Alzheimer’s Disease Progression (P2-9.003). In Neurology (Vol. 102, No. 7). Hagerstown, MD: Lippincott Williams & Wilkins, 2024, 5255.

[18] St Sauver J , Fu S , Sohn S , et al. Identification of delirium from real-world electronic health record clinical notes. J Clin Transl Sci 2023;7:e187. 37745932 10.1017/cts.2023.610 PMC10514685

[19] Sivarajkumar S , Tam TYC , Mohammad HA , et al. Extraction of sleep information from clinical notes of Alzheimer’s disease patients using natural language processing. J Am Med Inform Assoc 2024;31:2217–2227. 39001795 10.1093/jamia/ocae177 PMC11413436

[20] Suffoletto B , Zeigler A. Risk and protective factors for repeated overdose after opioid overdose survival. Drug Alcohol Depen 2020;209:107890. 10.1016/j.drugalcdep.2020.107890 PMC7127977 32058246

[21] Inouye SK , Westendorp RGJ , Saczynski JS. Delirium in elderly people. Lancet 2013;383:911. 23992774 10.1016/S0140-6736(13)60688-1 PMC4120864

[22] Inouye SK , et al. Clarifying confusion: the confusion assessment method: a new method for detection of delirium. Ann Intern Med 1990;113:941–948. 2240918 10.7326/0003-4819-113-12-941

[23] Fu S , Lopes GS , Pagali SR , et al. Ascertainment of delirium status using natural language processing from electronic health records. J Gerontol A Biol Sci Med Sci 2022;77:524–530. 35239951 10.1093/gerona/glaa275 PMC8893184

[24] Fu S , Jia H , Vassilaki M , et al. FedFSA: Hybrid and federated framework for functional status ascertainment across institutions. J Biomed Inform. 2024;152:104623. 38458578 10.1016/j.jbi.2024.104623 PMC11005095

[25] Damschroder LJ , Aron DC , Keith RE , Kirsh SR , Alexander JA , Lowery JC. Fostering implementation of health services research findings into practice: A consolidated framework for advancing implementation science. Implementation Sci 2009;4: 1–15. 10.1186/1748-5908-4-50 PMC2736161 19664226

[26] Damschroder LJ , Reardon CM , Widerquist MAO , Lowery J. The updated Consolidated Framework for Implementation Research based on user feedback. Implementation Sci 2022;17: 1–16. 10.1186/s13012-022-01245-0 PMC9617234 36309746

[27] Clusmann J , Kolbinger FR , Muti HS , et al. The future landscape of large language models in medicine. Commun Med 2023;3:141. 37816837 10.1038/s43856-023-00370-1 PMC10564921

[28] Miaojiao S , et al. Using a large language model for breast imaging reporting and data system classification and malignancy prediction to enhance breast ultrasound diagnosis: retrospective study. JMIR Med Inform 2025;13:e70924. 40498674 10.2196/70924 PMC12175873

