# Supplementary material for: Development and validation of natural language processing algorithms in the national ENACT network
Source: J Clin Transl Sci. 2025 Aug 22;9(1):e199. doi: 10.1017/cts.2025.10116 (PMC12444719; doi:10.1017/cts.2025.10116)
Supplement: Wang et al. supplementary material [file S2059866125101167sup001.docx]

**Supplemental Materials**

***NLP Tools***

We selected the Open Health Natural Language Processing (OHNLP) Toolkit [[1]](https://paperpile.com/c/O2zglD/77Wv6), developed by the OHNLP consortium, as our primary NLP tool for extracting entities from clinical notes. The OHNLP Toolkit employs an ontology-driven, dictionary-based approach with MedTagger[[2]](https://paperpile.com/c/O2zglD/8V3cJ) as the core entity extraction component. The Toolkit is highly adaptable, accepting input from local file systems, relational databases, and the Epic Clarity reporting database, and delivering output to file systems and relational databases. Compared to other NLP tools, such as MetaMap[[3]](https://paperpile.com/c/O2zglD/jH3QC), the Toolkit offers higher throughput, better customization, and increased scalability, making it ideal for processing large volumes of notes. Its effectiveness has been demonstrated in large EHR data projects, such as the National COVID Cohort Collaborative (N3C)[[4]](https://paperpile.com/c/O2zglD/Pug33). Furthermore, we partnered with the OHNLP development team and created a dedicated support channel on Slack to assist sites with troubleshooting during validation and deployment.

The OHNLP Toolkit is built using the Java programming language. Recognizing that some sites do not use Java, we are developing a Python alternative that uses SpaCy[[5]](https://paperpile.com/c/O2zglD/XE4Ik) and can be deployed on servers running the Linux, macOS, and Windows operating systems. Like the Toolkit, the Python alternative will accept input from various sources, including file systems, .csv files, .zip archives, and relational databases, and deliver output to file systems and relational databases. Another key feature is that it will be compatible with the Toolkit rulesets, which enables smooth switching between the two tools without losing functionality. We plan to make the Python alternative publicly available through the OHNLP consortium’s GitHub repository [6], ensuring it is freely accessible to the informatics and biomedical research communities.

***Extension of the Common Data Model for NLP-derived Data***

The ENACT network uses the ACT/i2b2 common data model (CDM)[[6]](https://paperpile.com/c/O2zglD/ZeCwA) to harmonize the EHR data (more recently, the OMOP CDM has been compatible with ENACT; in the future, the PCORnet CDM will also be compatible). In the ACT/i2b2 CDM, a concept is a standardized term from a medical terminology (e.g., ICD-10-CM) or a custom terminology (e.g., a hospital’s in-house oncology terminology), and a fact is a particular instance or observation of a concept for a specific patient (e.g., ICD-10-CM:S92.4). NLP-derived entities or facts are stored in the i2b2 *observation_fact* table, which also stores structured EHR data. Detailed documentation on concepts and facts and their representations in the *observation_fact* table are available at the i2b2 Wiki[[7]](https://paperpile.com/c/O2zglD/otx3v). The Working Group established a set of conventions for representing NLP-derived entities in the *observation_fact* table outlined in Supplement Table 1. The conventions are grounded in a philosophy that emphasizes scalability and customization, enabling them to meet the diverse needs of various sites and projects. Recognizing that each site may have unique requirements and each project may include differing granularity of NLP-derived data, the conventions are designed to be flexible, allowing for adjustments and extensions as needed. By prioritizing adaptability, these conventions support the broad applicability and long-term sustainability of NLP in the ENACT network.

The CONCEPT_CD field in the *observation_fact* table is the key field to store NLP-derived entities. Because structured EHR data is also stored in the *observation_fact* table, it is critical to distinguish NLP-derived data from them. To accomplish this, NLP-derived entities are prefixed with the string “NLP.” NLP-derived entities are translated to concepts obtained from standardized medical terminologies such as the ICD-10-CM or a project’s custom terminology. When using a concept from a standard terminology, the entry in the CONCEPT_CD field in the *observation_fact* table is formatted as NLP|<PROJECT_NUM>|<STANDARD_VOCABULARY_PREFIX>:<STANDARD_CONCEPT_CODE>, where <STANDARD_VOCABULARY_PREFIX> indicates the standard terminology used and <STANDARD_CONCEPT_CODE> represents the concept in the standard terminology. For example, the entity “fracture of a great toe” extracted by an NLP algorithm is represented as NLP|001|ICD-10-CM:S92.4 in the CONCEPT_CD field. When using a concept from a custom terminology, the entry in the CONCEPT_CD field is formatted as NLP|<PROJECT_NUM>|CUSTOM|<PROJECT_NAME>:<CONCEPT>, where <PROJECT_NAME> specifies the project, and <CONCEPT> describes the custom concept. For example, the entity “snoring” extracted by an NLP algorithm in a project on sleep is represented as NLP|001|CUSTOM|SLEEP:SNORING in the CONCEPT_CD field. **Supplement Table 1** provides additional examples.

In addition to the main entity, NLP algorithms often output contextual information, with most using the ConText algorithm[[8]](https://paperpile.com/c/O2zglD/Sc46n), which includes attributes such as *experiencer*, *certainty*, and *temporality*. The OHNLP Toolkit also employs the ConText algorithm to extract contextual information. The MODIFIER_CD field in the *observation_fact* table is used to store contextual attributes, formatted as NLP|<ATTRIBUTE>:<VALUE> (e.g., NLP|EXPERIENCER:PATIENT). A single NLP-derived entity may be associated with multiple contextual attributes, requiring multiple MODIFIER_CD entries. This could cause the *observation_fact* table to become excessively large, potentially consuming a significant amount of storage. However, this limitation is offset by the enormous flexibility of the entity-attribute-value table design in i2b2, which allows it to store any type of NLP-derived data without requiring custom database tables. For example, an NLP-derived medication entity may include contextual attributes related to dose. The MODIFIER_CD field, together with VALTYPE_CD, TVAL_CHAR, and NVAL_NUM, is used to store numeric attributes such as “325 mg QD PO” associated with the CONCEPT_ID entry “aspirin” (see Supplement Table 1).

Sometimes, text snippets like phrases or sentences related to an NLP-derived entity may be useful for research; such text snippets are stored in the OBSERVATION_BLOB field in the *observation_fact* table. However, populating text snippets is optional since they may inadvertently contain protected health information, and ENACT's current policy allows only limited datasets in the network’s data repositories. Beyond phrases or sentences, other types of information, such as location data, may also be stored in the OBSERVATION_BLOB field.

The Working Group has proposed adding two new columns to the *observation_fact* table to store the cohort definition and note type. A key benefit of the ENACT network is the consistent definition of cohorts, as each cohort definition query is associated with a unique Query ID that is propagated throughout the network. This unique Query ID can unambiguously identify the cohort on which an NLP algorithm was run. Standard terminologies for note type are available, for example, in SNOMED CT and LOINC (e.g., STD|LOINC:59258-4 denotes a standard document type according to LOINC Note Type). Local custom terminologies may also be used (e.g., CUS|[local note type]). For a project, sites typically agree on the specific note type from which to extract NLP data.

**Supplement Table 1. Conventions for using the i2b2 *observation_fact* table for NLP-derived data.**

| 1. NLP-derived data:    1. To distinguish NLP-derived data from other data types, prefix the entry in the CONCEPT_CD field with “NLP.”    2. When using a concept from a standard terminology, format the entry in the CONCEPT_CD field as NLP\|<PROJECT_NUMBER>\|<STANDARD_VOCABULARY_PREFIX>: <STANDARD_CONCEPT_CODE>. The <STANDARD_VOCABULARY_PREFIX> indicates the standard terminology used, and <STANDARD_CONCEPT_CODE> represents the concept in the standard terminology.   Examples:  NLP\|001\|ICD-10-CM:S92.4  NLP\|001\|MSH:D012890  NLP\|001\|SNOMED:258158006  NLP\|001\|OMOP:4248728  NLP\|001\|UMLS:C0037313   - 1. When using a concept from a custom terminology, format the entry in the CONCEPT_CD as NLP\|<PROJECT_NUMBER>\|CUSTOM\|<PROJECT_NAME>:<CONCEPT>. The <PROJECT_NAME> specifies the project, and <CONCEPT> describes the custom concept.   Example:  NLP\|001\|CUSTOM\|SLEEP:SNORING for the project “SLEEP” with the custom concept "SNORING."   1. Contextual information:    1. Store contextual information for NLP-derived data in the MODIFIER_CD field. When using attributes output by the ConText algorithm, format the entry in the MODIFIER_CD field as NLP\|<ATTRIBUTE>:<VALUE>.   Examples:  NLP\|EXPERIENCER:PATIENT  NLP\|EXPERIENCER:OTHER  NLP\|CERTAINTY:NEGATED  NLP\|CERTAINTY:HYPOTHETICAL  NLP\|CERTAINTY:POSITIVE  NLP\|TEMPORALITY:PRESENT  NLP\|TEMPORALITY:HISTORYOF   - 1. For additional values beyond those output by the ConText algorithm, format them to adhere to the established naming convention.   Example:  NLP\|EXPERIENCER:FATHER   \| CONCEPT_CD \| MODIFIER_CD \| VALTYPE_CD \| TVAL_CHAR \| NVAL_NUM \| \| --- \| --- \| --- \| --- \| --- \| \| NLP\|001\|CUSTOM\|SLEEP: SNORING \| @* \|  \|  \|  \| \| NLP\|001\|CUSTOM\|SLEEP: SNORING \| NLP\|EXPERIENCER:PATIENT \|  \|  \|  \| \| NLP\|001\|CUSTOM\|SLEEP: SNORING \| NLP\|TEMPORALITY:HISTORY_OF \|  \|  \|  \| \| NLP\|001\|CUSTOM\|SLEEP: SNORING \| NLP\|CERTAINTY:POSITIVE \|  \|  \|  \|   *Built as default   1. Additional numeric information:    1. For additional numeric data like medication dose, use the MODIFIER_CD field with VALTYPE_CD, TVAL_CHAR, and NVAL_NUM fields.   Example: “325 mg QD PO” associated with the CONCEPT_ID entry “aspirin”   \| CONCEPT_CD \| MODIFIER_CD \| VALTYPE_CD \| TVAL_CHAR \| NVAL_NUM \| \| --- \| --- \| --- \| --- \| --- \| \| NLP\|CUSTOM\|MEDEX: ASPIRIN \| @ \| <null> \| <null> \| <null> \| \| NLP\|CUSTOM\|MEDEX: ASPIRIN \| NLP\|MED:DOSE \| N \| E \| 325 \| \| NLP\|CUSTOM\|MEDEX: ASPIRIN \| NLP\|MED:FREQ \| T \| QD \| <null> \| \| NLP\|CUSTOM\|MEDEX: ASPIRIN \| NLP\|MED:ROUTE \| T \| PO \| <null> \|  1. Text snippets (optional):    1. For text snippets like phrases or sentences related to an NLP-derived datum, use   the OBSERVATION_BLOB field typically with MODIFIER_CD = @.  Example:   \| CONCEPT_CD \| MODIFIER_CD \| OBSERVATION_BLOB \| \| --- \| --- \| --- \| \| NLP\|001\|CUSTOM\|SLEEP: SNORING \| @ \| “*husband admits she snored in the past on occasion*” \|  1. Cohort definition and note type^[note]^:    1. Two additional columns can be added to the table:       1. Cohort Definition: Document using the ENACT Network Query ID.       2. Note Type: Use SNOMED CT/LOINC Note Type values.   Example convention: STD\|LOINC:59258-4, or CUS\|[local note type]. |
| --- | --- | --- | --- | --- | --- | --- | --- | --- | --- | --- | --- | --- | --- | --- | --- | --- | --- | --- | --- | --- | --- | --- | --- | --- | --- | --- | --- | --- | --- | --- | --- | --- | --- | --- | --- | --- | --- | --- | --- | --- | --- | --- | --- | --- | --- | --- | --- | --- | --- | --- | --- | --- | --- | --- | --- | --- |

[note]: the note type standardization is still under discussion and hasn’t been finalized.

***Extension of the Ontology for NLP-derived Data***

To query the NLP-derived data in the ENACT network, the Working Group has extended the ACT ontologies to include concepts that represent NLP-derived entities. The ontologies for NLP-derived data are visually distinct from those for structured EHR data in the SHRINE interface. Two new ontologies have been developed. The first ontology, Note Types, enables the identification of types of clinical notes and is based on LOINC’s document ontology. The current LOINC document ontology describes key attributes of clinical documents in five axes: type of service, kind of document, setting, role, and subject matter domain, with each axis having a set of controlled terms. The second ontology, Clinical Concepts by Project, provides project-specific concepts for querying task-specific NLP-derived entities. Each project-specific ontology is provided as a separate subtree, and concepts can be derived from standard terminologies, such as ICD-10, or a custom terminology developed specifically for the project. For example, the Rare Disease ontology contains concepts from the Orphanet Rare Disease Ontology (ORDO), whereas the Sleep Ontology utilizes custom concepts such as DAY_SLEEP and SLEEP_PROBLEMS. **Supplement Figure 1** provides a screenshot of the current ENACT NLP ontologies.

Whenever possible, project-specific ontologies are designed to leverage standard vocabularies, enabling the reuse and comparison of entities derived from both structured EHR data and clinical notes. Contextual information stored in the CONCEPT_CD field and additional information stored in the OBSERVATION_BLOB field are currently not queryable through the SHRINE interface, but data stored in the CONCEPT_CD field can be queried via the i2b2 interface.


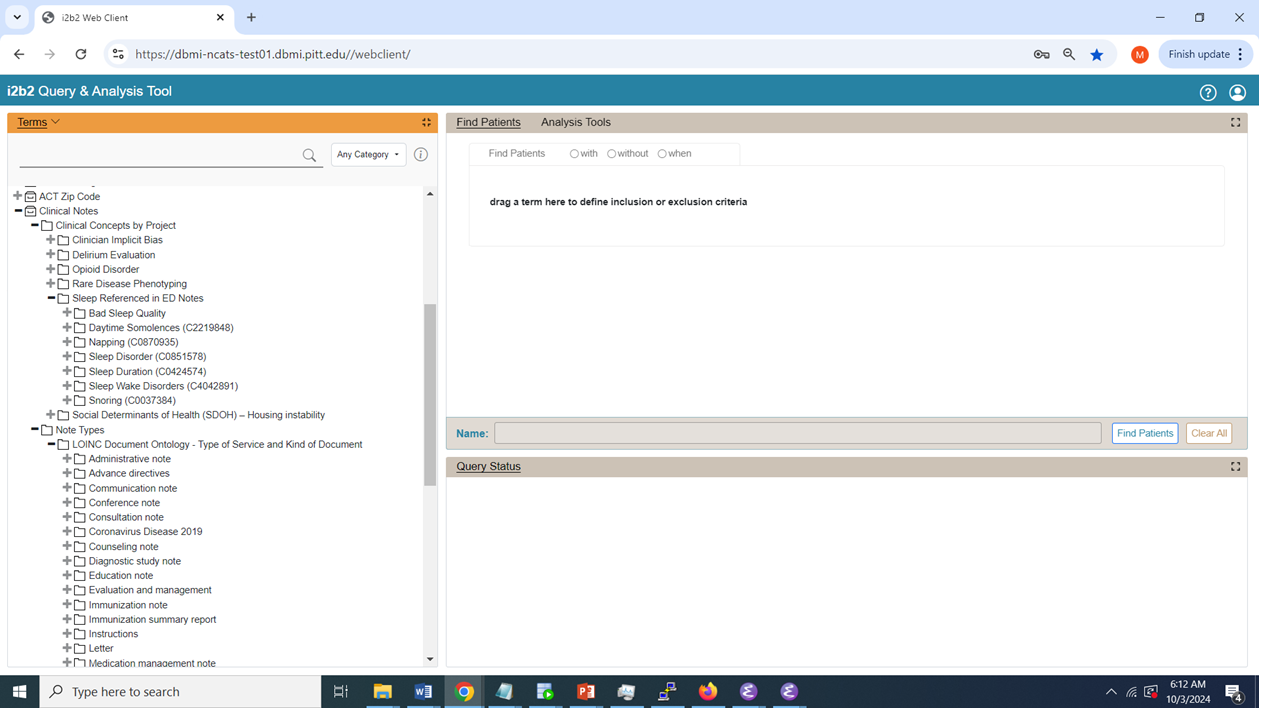


Supplement Figure 1. ENACT NLP ontology implementation.

***Evaluation of NLP Algorithms***

We developed an approach for evaluating NLP algorithms that accommodates the federated nature of the ENACT network, where clinical notes from multiple institutions cannot be aggregated at a central location. Consequently, the evaluation framework emphasizes both intra-site and cross-site validation, facilitated by explicitly shared definitions of cohort specification and applicable note types. This gold standard is manually derived by development sites, and a computable silver standard is established that requires minimal manual review by deployment sites. **Supplement Figure 2** provides an overview of this approach.


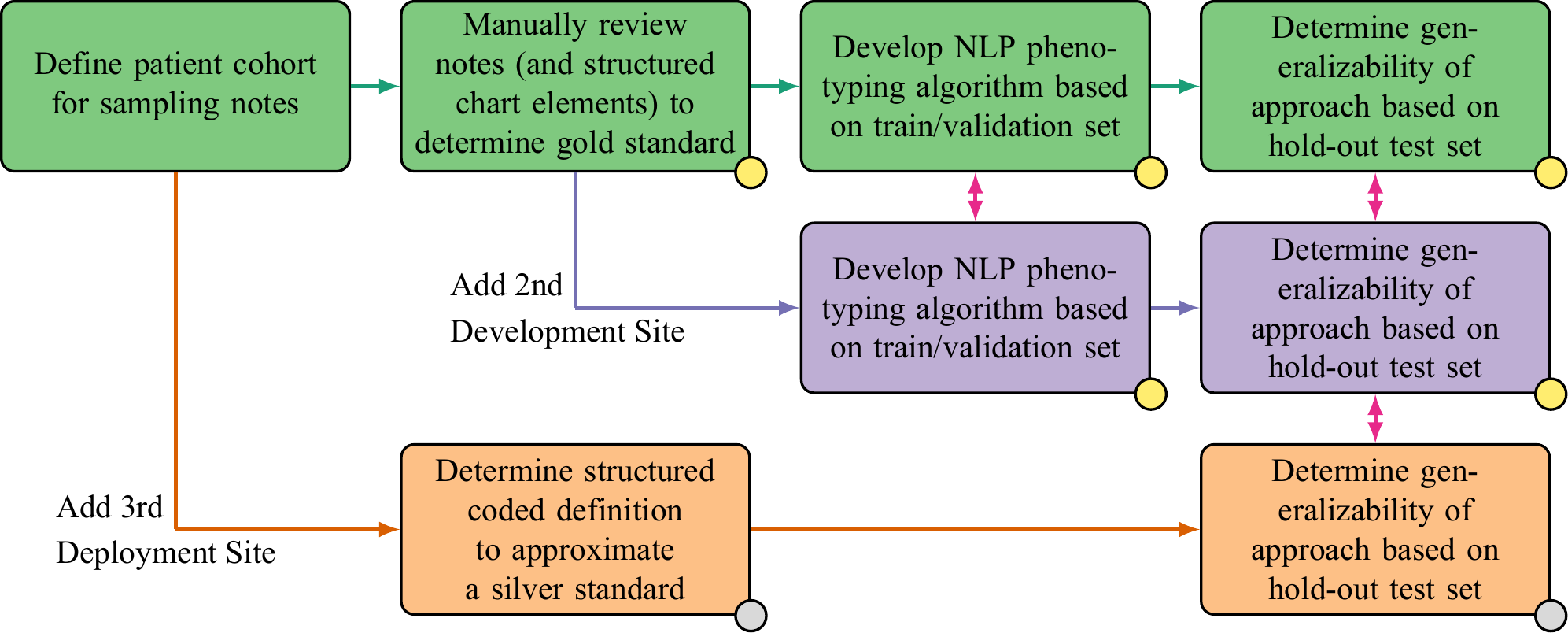


Supplement Figure 2. Evaluation of NLP algorithms across development and deployment sites in the national ENACT network.

*The green, purple, and orange boxes indicates tasks undertaken at each of the three types of sites, respectively. The tasks associated with a gold standard have a gold circle and the tasks associated with a silver standard have a silver circle. The red arrows between sites indicate the possibility of a cross-site evaluation or comparison.

Specifications for an algorithm typically begin with a development site that defines a cohort, a relevant note type, and a method for sampling the notes when a large volume is available. A second development site reviews and adapts these specifications for generalizability; for example, they may clarify the definition of the note type and the sampling procedure. Once the specifications are finalized, the development sites employ a manual review process to establish a gold standard reference for the NLP algorithm. In conjunction with this gold standard reference, the sites mutually agree upon a silver standard reference, which can be derived from structured data alone or with minimal additional manual review. The development sites iteratively refine the algorithm, allowing for comparisons of rules, models, and, most critically, unforeseen challenges.

Once the algorithm development is completed, it is shared with the Working Group and the validation sites. In rare cases, the algorithm cannot be shared among sites due to privacy concerns; in such instances, a detailed training method is provided, enabling each site to construct the algorithm using its data. The development sites assess the algorithm using both gold and silver standard references. They compare the performance of these two standards to ensure that the silver standard is adequate for broader evaluation at other sites. Finally, the validation sites test the algorithm at each of their locations using the readily computable silver standard reference.


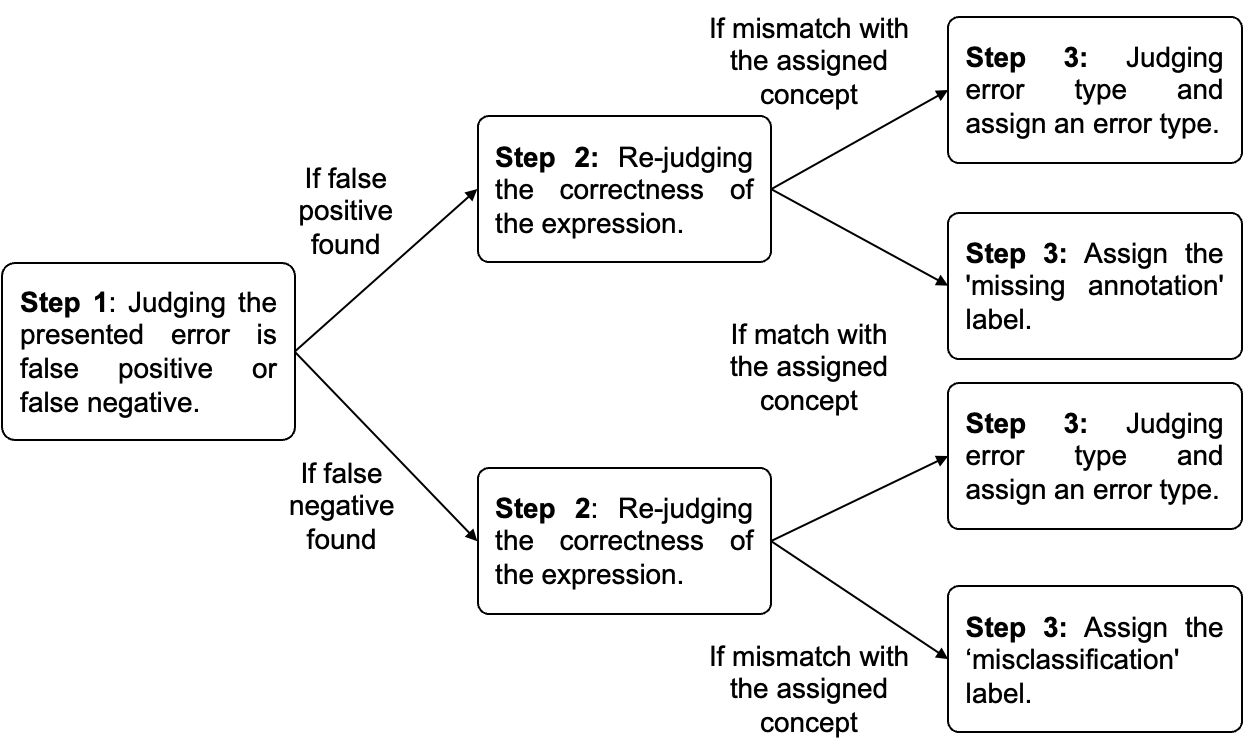


Supplement Figure 3 Overview of the logic and process for multisite error analysis

This evaluation approach lowers the barrier to cross-site validation of NLP algorithms by establishing a consistent and replicable framework for effective collaboration among sites. The workflow shown in **Supplement Figure 3** provides a clear path for sites to evaluate algorithms in their environments, which may differ slightly from those at the development sites. This approach minimizes the need for extensive manual interventions, accelerates the validation process, and promotes broader adoption of NLP algorithms across multiple sites. As cross-site validation becomes increasingly efficient, streamlined, and scalable, NLP algorithms can be feasibly deployed across the entire ENACT network.

***Standardization of Error Analysis***

Error analysis is a common practice in the development and validation of NLP algorithms. **Supplement Figure 3** presents an overview of the site-level error analysis process adopted by the Working Group. We used our previously created error taxonomy and the clinical Text Retrieval and Use towards Scientific rigor and Transparent (TRUST) process [[9]](https://paperpile.com/c/O2zglD/TCCyX) to guide and standardize the error analysis. We focused on identifying the four most common error types: logic errors, linguistic errors, contextual errors, and annotation errors. We also gathered information about the task, the focus area (e.g., disease, medication class, or SDoH category), site expertise in clinical NLP, the NLP methodology, and annotation details. We utilized error analyses during algorithm development and applied the results to enhance performance, as well as after the algorithm's development to collect data on its generalizability, portability, and limitations.

**Supplement Table 2. Site-Specific Data Sources and Preprocessing Challenges in ENACT NLP Implementation**

| Site | Primary EHR System(s) | Data Access Source | Template/Format Challenges | Real-World Examples |
| --- | --- | --- | --- | --- |
| University of Pittsburgh | Epic & Cerner | CDW/Epic Clarity | Mixed vendor templates; legacy Cerner data integration | Radiology reports vary by imaging modality; some structured data embedded in narrative text; notes duplications |
| University of Kentucky | Epic | Epic Clarity reporting database | EHR transition complexity (pre/post-2021) | Nursing assessments stored as free text despite structured template availability |
| University of California Los Angles | Epic | Epic Clarity | Template evolution over time | Radiology impression sections: older studies narrative vs. newer structured reporting |
| UT Southwestern | Epic & OMOP | OMOP notes table | Dual system integration | Risk screening templates exported as unstructured text blocks |
| Scripps Research | Epic | Epic Clarity | Template changes over time; standardization | Some structured data stored as free text (despite structured template availability) |
| UT Health Science Center | Epic | OMOP note table & EHR direct access | Template standardization issues | HPI narratives, geriatric assessment templates, nursing flow sheets saved as notes |
| Medical University of South Carolina | Epic | custom RDW (Research Data Warehouse) and OMOP note table | Simple plain text formatting and structurally significant newlines removed as a consequence of Epic ETL to RDW | Poorly encoded characters (e.g., using Windows-1250 code page but flagged as ASCII or Unicode) can cause NLP to fail. Tables (e.g., of labs or medications) that are correctly formatted when viewed in Epic have their whitespace or rich text based formatting stripped so the NLP engine does not have access to this additional information organization. |
| University of Alabama Birmingham | Cerner | CDW[[10]](https://paperpile.com/c/O2zglD/afE9), Custom staging “Medics” Table | Multiple note formats, minimal templating | Structured data embedding in narrative text, PDF conversion of DownTime notes |
| Mayo Clinic | Epic | CDW | Multi-site template variations | Structured medication data occasionally documented in progress notes |
| Weill Cornell Medicine | OMOP | OMOP notes table | Variations in referral notes | Referral notes encompass a variety of document types, including progress notes, structured EHR, radiology reports, and others. |
| University of Rochester Medical Center | Epic & OMOP | OMOP NOTE table | Inconsistent note structure and template | Various note formats are found among different note types (e.g. Comprehensive Assessment, Provider Consult, etc.); Note templates are not consistently utilized, lots of data are documented as free text. Note structures are highly customized by care teams and changed overtime |

**References**

1. [**Wen A, Fu S, Moon S, *et al.*** Desiderata for delivering NLP to accelerate healthcare AI advancement and a Mayo Clinic NLP-as-a-service implementation. *npj Digital Medicine* Nature Publishing Group, 2019; **2**(1): 1–7.](http://paperpile.com/b/O2zglD/77Wv6)

2. [**Liu H, Bielinski SJ, Sohn S, *et al.*** An information extraction framework for cohort identification using electronic health records. *AMIA Joint Summits on Translational Science proceedings. AMIA Joint Summits on Translational Science* 2013; **2013**: 149–153.](http://paperpile.com/b/O2zglD/8V3cJ)

3. [**Aronson AR**. Effective mapping of biomedical text to the UMLS Metathesaurus: the MetaMap program. *Proceedings: a conference of the American Medical Informatics Association / ... AMIA Annual Fall Symposium. AMIA Fall Symposium* American Medical Informatics Association, 2001; : 17.](http://paperpile.com/b/O2zglD/jH3QC)

4. [**Liu S, Wen A, Wang L, *et al.*** An open natural language processing (NLP) framework for EHR-based clinical research: a case demonstration using the National COVID Cohort Collaborative (N3C). *Journal of the American Medical Informatics Association: JAMIA* 2023; **30**(12): 2036–2040.](http://paperpile.com/b/O2zglD/Pug33)

5. [***spaCy 2: Natural language understanding with Bloom embeddings, convolutional neural networks and incremental parsing***. *Sentometrics Research*. 2017 [cited September 23, 2024]. (](http://paperpile.com/b/O2zglD/XE4Ik)<https://sentometrics-research.com/publication/72/>[).](http://paperpile.com/b/O2zglD/XE4Ik)

6. [**Klann JG, Abend A, Raghavan VA, Mandl KD, Murphy SN**. Data interchange using i2b2. *Journal of the American Medical Informatics Association: JAMIA* 2016; **23**(5): 909–915.](http://paperpile.com/b/O2zglD/ZeCwA)

7. [**(jmt67) UU**. *OBSERVATION_FACT Table - Server (Cells) Design - i2b2 Community Wiki*. [cited September 25, 2024]. (](http://paperpile.com/b/O2zglD/otx3v)<https://community.i2b2.org/wiki/display/ServerSideDesign/OBSERVATION_FACT+Table>[).](http://paperpile.com/b/O2zglD/otx3v)

8. [**Harkema H, Dowling JN, Thornblade T, Chapman WW**. ConText: an algorithm for determining negation, experiencer, and temporal status from clinical reports. *Journal of biomedical informatics* 2009; **42**(5): 839–851.](http://paperpile.com/b/O2zglD/Sc46n)

9. [**Fu S, Wang L, He H, *et al.*** A taxonomy for advancing systematic error analysis in multi-site electronic health record-based clinical concept extraction. *Journal of the American Medical Informatics Association : JAMIA* 2024; **31**(7): 1493–1502.](http://paperpile.com/b/O2zglD/TCCyX)

10. [**Osborne JD, Khare A, Dempsey DM, *et al.*** Phenotype Detection Registry System (PheDRS)-Implementation of a Generalizable Single Institution Clinical Registry Architecture. *AMIA Annual Symposium Proceedings* 2018; **2018**Published online: 2018.](http://paperpile.com/b/O2zglD/afE9)
